# Maternal Satisfaction with Intrapartum Nursing Care and Its Associated Factors among Mothers Who Gave Birth in Public Hospitals of North Wollo Zone, Northeast Ethiopia: Institution-Based Cross-Sectional Study

**DOI:** 10.1155/2020/8279372

**Published:** 2020-04-27

**Authors:** Asmamaw Demis, Ribka Nigatu, Derebe Assefa, Getnet Gedefaw

**Affiliations:** ^1^Department of Nursing, College of Health Sciences, Woldia University, P.O. Box: 400, Woldia, Ethiopia; ^2^Department of Midwifery, College of Health Sciences, Woldia University, P.O.Box:400, Woldia, Ethiopia

## Abstract

**Background:**

Now a day, satisfaction had been identified as the major index to assess the quality of health-care provision in the world including Ethiopia. Mothers judge the quality of intrapartum care received based on their satisfaction with the services provided, thus influencing their utilization of the available health facilities. Therefore, this study aimed to assess maternal satisfaction with intrapartum care and associated factors among mothers who gave birth in public hospitals in North Wollo Zone, Northeastern Ethiopia, 2019.

**Methods:**

Institutional-based cross-sectional quantitative study was conducted in public hospitals of North Wollo Zone, and a total of 398 study participants were selected by using a systematic random sampling method. Data was collected using a standardized questionnaire by direct interviewing of study participants, and data was analyzed using SPSS 24 versions to determine the frequency of variables. Logistic regression was carried out to identify factors associated with maternal satisfaction.

**Results:**

From the total of 398 study participants, about 51% of women were satisfied with the hospital-based intrapartum nursing care. Being rural in residency (AOR: 2.03; 95% CI: 1.05-3.93), time to be seen by health-care providers (AOR: 2.82; 95% CI: 1.46-5.46), having history of ANC follow-up (AOR: 3.73; 95% CI: 1.12-12.57), and getting adequate meal (AOR: 3.96; 95% CI: 1.13-13.83) had showed statistical significant association with maternal satisfaction.

**Conclusion:**

In this study, the overall maternal satisfaction with intrapartum nursing care was low. Therefore, improving ANC follow-up, early examined by health-care providers, and getting adequate meal while in labour and delivery might enhance women satisfaction with intrapartum nursing care services.

## 1. Introduction

Intrapartum nursing care is the care given by nurses and midwives for labouring mother during labour and delivery [1]. Even though the primary target of the United Nations' Sustainable Development Goal (SDG3) is to reduce the global maternal mortality rate to less than 70 per 100,000 live births, the quality of intrapartum care in most low- and middle-income countries was chronically poor, and this had been identified as one of the precursors to the unacceptably high maternal mortality rate in low- and middle-income countries [1]. The World Health Organization reported that approximately more than eight hundred fifty women die from preventable causes related to childbirth every day, with 99% of all these maternal deaths occurring in low- and middle-income countries [2].

Maternal satisfaction with intrapartum nursing care measures the ability of services to meet consumers' expectations, and it is an important determinant of the choice of health facility and its future utilization for labour and delivery services [3–7]. In Ethiopia, there are increasing needs of client-centered care and had been a growing consensus that patient service quality perceptions are critical for maintaining and monitoring the quality of health care [8–10]. According to WHO, more than 600,000 women die each year from complications arising from pregnancy and labour and delivery. The majority of maternal mortality occurred in sub-Saharan Africa (162, 000) and Southern Asia (83, 000) with these two regions accounted for 85% of global burden, with sub-Saharan Africa alone accounting for 56%. The estimated pregnancy-related mortality ratio was 412 deaths per 100,000 live births in Ethiopia [2, 11].

Different studies conducted in developed and developing countries including Ethiopia showed that ANC follow-up, educational status, waiting time, availability of basic drugs, cleanliness of the environment, delivery room and wards, cost paid to service and waiting area, privacy, educational status, and health providers' technical competence were the major factors associated with maternal satisfaction with intrapartum nursing care [8, 12–19].

In Ethiopia, among the total live births in the 5 years preceding the survey, 50% were delivered by a skilled provider and 48% were delivered in a health facility [20]. Only, provision of maternal health service does not improve maternal health; as a result, the World Health Organization promotes skilled attendance at every birth to reduce maternal mortality and recommends that women's satisfaction is the most important index to improve the quality and effectiveness of health-care provision [21]. Now a day, most patients in our country and specifically in public hospitals of North Wollo Zone complain about hospital services, particularly on delivery services. Despite having many studies done elsewhere, there is a paucity of data concerning maternal satisfaction with intrapartum nursing care in Ethiopia; particularly, there is a dearth of study in Northeastern Ethiopia. Therefore, this study aimed to assess mothers' satisfaction with intrapartum care and factors associated with it among mothers who gave birth at a public hospital in North Wollo Zone, Northeastern Ethiopia.

### 1.1. Objectives

#### 1.1.1. Specific Objectives

- To determine the magnitude of maternal satisfaction with intrapartum nursing care among mothers who gave birth in public hospitals of North Wollo Zone, Northeast Ethiopia.
- To identify factors associated with mothers satisfaction with intrapartum nursing care among mothers who gave birth in public hospitals of North Wollo Zone, Northeast Ethiopia.

## 2. Methods and Materials

### 2.1. Study Setting, Design, and Period

An institution-based cross-sectional quantitative study design was carried out at public hospitals in North Wollo Zone, Amhara National Regional State from January 01 to February 30, 2019. North Wollo Zone is found in Amhara region with a capital city of Woldia that is found 521 km away from Addis Ababa and 360 km form Bahirdar. Based on the 2007 Census conducted by the Central Statistical Agency of Ethiopia [22], the total population of North Wollo Zone was 1,500,303, an increase of 19.04% over the 1994 census, of whom 752,895 are men and 747,408 women, respectively. There are five public hospitals in North Wollo Zone, namely, Woldia General Hospital, Kobo Primary Hospital, Lalibela Primary Hospital, Meket Primary Hospital, and Wadila Primary Hospital. The hospitals are open for 24 hours in a day to provide curative, emergency, maternal, and child health services. Regarding health services, there are six hospitals, sixty-five health centers, and two hundred seventy-five health posts providing services to the community according to North Wollo Zone Health Office. Annual report from North Wollo Zone Health Office in 2019 indicated that the health coverage of institutional delivery was 78%.

### 2.2. Source Population and Study Population

The source population were all women who visited public hospitals in North Wollo Zone for delivery service, and all systematically selected women who gave birth at public hospitals during the study period were the study population.

### 2.3. Inclusion and Exclusion Criteria

Women who gave birth in the selected hospitals and discharged from postnatal ward during the data collection period were included, whereas women who were seriously ill during the study period were excluded from the study.

### 2.4. Sample Size and Sampling Procedures

The sample size was calculated using single population proportion formula with the assumption of 95% confidence interval, 5% margin of error, 61.9% of maternal satisfaction [14], and 10% of nonresponse rate. The final sample size for the study was found to be 398. There are five public hospitals in North Wollo Zone; among these, three hospitals were selected purposefully based on service provision to the public and provision of basic obstetrics and newborn care. Numbers of study subjects in each hospital were determined by proportion to population size from reviewing the average three-month delivery service report. The study participants were selected by systematic sampling method when the mothers were discharged from postnatal unit, and exit interview was performed after the delivery period.

### 2.5. Variables and Measurements

*Satisfaction*: the overall maternal satisfaction was measured based on the answer for satisfaction related question using a five point Likert scale, and the mean score of satisfaction was 64.66. Above the mean score of satisfaction were considered as satisfied “yes,” and below the mean as dissatisfied “no” [18]. During analysis, the responses of “very satisfied” and “satisfied” were classified as satisfied and responses of “very dissatisfied,” “dissatisfied,” and “neutral” as unsatisfied. For the overall satisfaction level, those who were satisfied in greater or equal to the mean score of the items were categorized under satisfied, and those who were satisfied in less than mean score of the items were categorized as unsatisfied.

*Waiting time*: the time between admissions to the time seen by health-care professional.

*Privacy:* the state of being free from being observed or disturbed by other people.

### 2.6. Data Collection Tool and Techniques

The data was collected by structured questionnaire which have three parts. The first part asks about sociodemographic information of mothers, and the second part is all about obstetric factors of the mother. Finally, the satisfaction of mothers was measured using questions which were adopted from Donabedian quality assessment framework [23] presented using a 5-point Likert scale ranging from very dissatisfied to very satisfied. The first draft of the English questionnaire was translated to Amharic language by independent translators then back to English language to check for consistency. Privacy and confidentiality were assured by not writing the name of the study subjects.

### 2.7. Data Quality Control

Two days training were given for data collectors and supervisors on how to ask and fill the questionnaire and how to approach the respondents. On each data collection day, the collected data were reviewed and checked for mistakes, legibility of handwriting, completeness, and consistency, and any mistake or ambiguity were cleared by principal investigator and supervisor; any problems faced in the time of data collection were discussed, and immediate solution was taken. The questionnaire was pretested on 5% of sample size at Wadila Primary Hospital before the actual data collection to see the accuracy of responses, language clarity, and appropriateness of the tools. The necessary amendments were done based on the findings of the pretest. The amended tools were used for actual data collection at the selected health facilities.

### 2.8. Data Processing and Analysis

The collected data were coded, cleaned, and entered into Epi data version 4.2 and exported to SPSS window version 24 for analysis. Bivariate analysis, crude odds ratio with 95% CI, was used to see the association between each independent variable and the outcome variable by using binary logistic regression. All variables with *P* ≤ 0.25 in the bivariable analysis were included in the final model of multivariable analysis in order to control all possible confounders. Adjusted odds ratio (AOR) with 95% CI were estimated to identify factors associated with mothers' satisfaction towards intrapartum nursing care using multivariable logistic regression analysis. Level of statistical significance was declared at *P* value < 0.05.

## 3. Results

### 3.1. Sociodemographic Characteristics of Respondents

A total of 398 delivering mothers from three public hospitals participated in the study, making a response rate of 100%. Of the total study participants, 171 (43%) of the women were from Woldia General Hospital, 113 (28.4%) were from Kobo Primary Hospital, and the rest 114 (28.6%) were from Lalibela Primary Hospital. The mean age of the mothers was 27.68 (±5.08 SD). About 59% of the women were unable to write and read, and the majority, 95.2%, were married. Two hundred sixty-five (66.6%) of study participants were housewives, and 298 (74.9%) of mothers came from urban areas. Regarding family size majority, 352 (88.4%) of the study subjects had less than or equal to four-family size (Table 1).

### 3.2. Obstetric Characteristics of Respondents

Regarding age at first pregnancy majority, 368 (92.5%) were married before the age of 18 years. Concerning gravidity and parity, 235 (59%) were primigravida and 276(69.3%) were primipara. Almost all, 397 (99.7%), had not experienced neonatal death, and 395 (99.2%) had not experienced a history of stillbirth. Concerning antenatal care follow-up, 375 (94.2%) had a history of ANC follow-up; of them, 230 (61.3%) had less than four visits. Regarding pregnancy status, 381 (95.7%) were wanted and majority, 365 (91.7%) mothers, delivered through spontaneous vaginal delivery (Table 2).

### 3.3. The Satisfaction of Labouring Mothers

**Figure 1:**
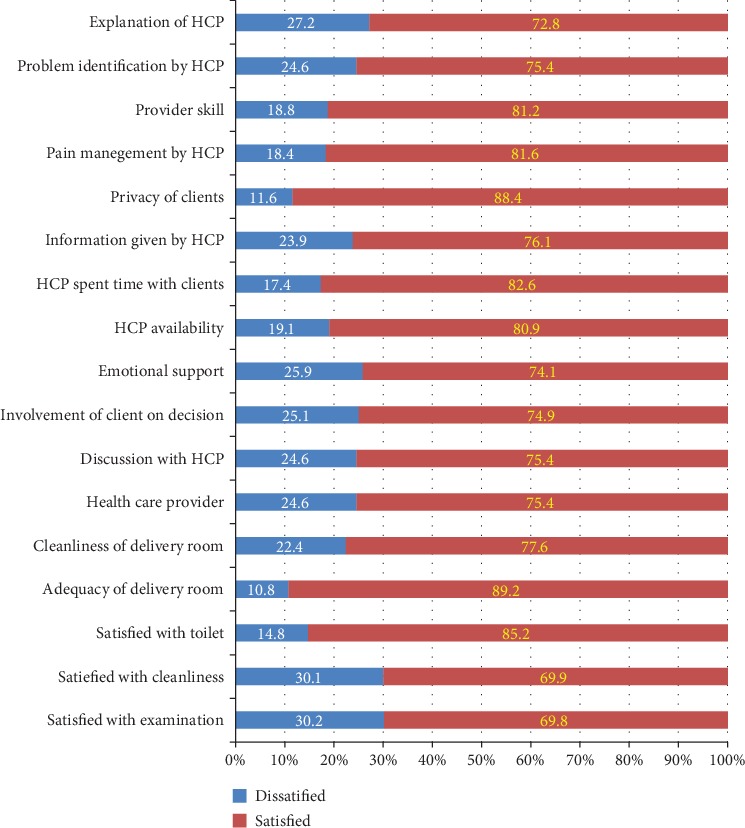
Maternal satisfaction among mothers who gave birth at public hospitals in North Wollo Zone, Northeast Ethiopia, 2019 (*n* = 398).

Among 398 study participants, 51.0% of respondents were satisfied by the provision of hospital overall intrapartum nursing care. Among 398 study participants, 69.8% of labouring women were satisfied with examination, 77.6% with cleanliness, 85.2% with toilet, 89.2% with adequacy of delivery room, 75.4% with problem identification of health-care provider, and 88.4% with privacy of clients (Figure 1).

### 3.4. Factors Associated with Maternal Satisfaction

Bivariate and multivariate logistic regressions were conducted to examine the association between dependent and independent variables. First, on bivariate logistic regression analysis, those variables that had a significant association with the dependent variables with *P* values of less than or equal to 0.25 were entered to multivariate logistic regression. In the multivariable model, residence, time to be seen by a health-care provider, ANC follow-up, and getting adequate meal had showed statistically significant association with maternal satisfaction on intrapartum nursing care.

Mothers who came from rural residents were almost two times more likely satisfied than urban residents (AOR: 2.03; 95% CI: 1.05-3.93). Mothers who were seen by health-care providers in less than 20 minutes were more likely satisfied as compared with the counterparts (AOR: 2.82; 95% CI: 1.46-5.46). Mothers who had not a history of ANC follow-up (AOR: 3.73; 95% CI: 1.12-12.57) and getting an adequate meal (AOR: 3.96; 95% CI: 1.13-13.83) were almost four times more likely satisfied as compared with their counterparts (Table 3).

## 4. Discussion

Generally, this study addresses the magnitude of maternal satisfaction with intrapartum nursing care and its associated factors among mothers who gave birth at North Wollo Zone public hospitals, Northeastern Ethiopia. Time to be seen with health-care providers, ANC follow-up, getting adequate meal, and residence were factors associated with maternal satisfaction with intrapartum nursing care.

This study revealed that the overall satisfaction of mothers on delivery service was found to be 51.0% (95% CI: 46.2%-56%), which is in line with a study conducted in Nepal (55.5%) [19] and Nairobi Kenya (56%) [24], higher than the study, which was conducted in Eritrea (20.8%) [25], Gondar Ethiopia (31.3%) [16], and Addis Ababa Ethiopia (19%) [9]. The possible deference may be due to improvement of intrapartum nursing care for labouring mothers due to high government concern for mothers and newborns to reduce maternal and neonatal mortality and morbidity. However, it was lower than a study conducted in Egypt (78.5%) [12], Wolaita Zone (82.9%) [26], Southern Ethiopia (90.2%) [27], Jimma University Specialized Hospital (77%) [28], Assella Hospital (80.7%) [29], Mekelle Ethiopia (79.7%) [30], Debre Markos town (81.7%) [15], and Felege Hiwot Referral Hospital Northwest Ethiopia (74.9%) [31]. This difference might be due to a real difference in the quality of services provided, the expectation of mothers, or the type of health facilities since most of them were primary hospitals.

In this study, being rural residents were almost two times more likely to be satisfied as compared with urban residents. This was in line with the study conducted in Wolaita Zone Ethiopia [26]. This might be due to mothers who came from rural residents had low expectation of different services and they became satisfied with the care they obtained at the intrapartum period. Similarly, those study participants who waited 20 minutes and less to be seen with health-care providers were 2.82 times more likely satisfied than their counterparts. This is in line with the study conducted in referral hospitals of Amhara region, Ethiopia [14], Assella Hospital [29]. This might be because long waiting time resulted in dissatisfaction due to poor cleanliness of toilet, the cost paid to service, and poor waiting area cleanliness and comfort.

Mothers who had not a history of ANC follow-up were 3.73 times more likely satisfied with intrapartum nursing care as compared with those mothers who had ANC follow-up which is supported with the study conducted in Assella Hospital [29] and Felege Hiwot Referral Hospital [31]. This might be due to the fact that getting awareness during counselling session of ANC follow-up and media exposure results in better expectation on quality of service greater than the real service which results in low satisfaction with intrapartum nursing care. Mothers getting adequate meals were almost four times more likely satisfied with intrapartum care as compared with their counterparts. This might be due to the fact that mothers who get a meal and other services which is similar to the service they get from their home results in better satisfaction.

### 4.1. Limitation of the Study

Findings might be subject to social desirability bias since interviews with mothers were conducted in the compounds of the health facilities.

## 5. Conclusion

In this study, the overall maternal satisfaction with intrapartum nursing care was low. Time to be seen with health-care providers, ANC follow-up, getting adequate meal, and residence were factors associated with maternal satisfaction with intrapartum care. Health-care providers should provide patient-centered care by fulfilling the mother's expectation as a crucial means to engage them with hospital delivery.

## Figures and Tables

**Table 1:** Sociodemographic characteristics of mothers who gave birth in public hospitals of North Wollo Zone, Northeastern Ethiopia, 2019 (*N* = 398).

Variables	Category	Frequency	Percentage
Age	≤24 years	160	40.2
	25-34 years	213	53.5
	≥35 years	25	6.2

Marital status	Married	379	95.2
	Widowed	14	3.5
	Others^∗^	5	1.3

Religion	Orthodox	329	82.7
	Muslim	58	14.6
	Others^∗∗^	11	2.7

Educational status	Unable to read and write	235	59.0
	Able to read and write	86	21.6
	Primary education	33	8.3
	Secondary education and above	44	11.1

Occupational status	Housewife	265	66.6
	Civil servant	109	27.4
	Private employee	24	6.0

Residence	Urban	298	74.9
	Rural	100	25.1

Family size	≤4	352	88.4
	>4	46	11.6

Monthly income	<2000 ETB	176	44.2
	≥2000 ETB	222	55.8

^∗^others (single and divorce); ^∗∗^others (Protestant, Catholic).

**Table 2:** Obstetric characteristic of mothers who gave birth at a public hospital in North Wollo Zone, Northeast Ethiopia, 2019 (*n* = 398).

Variables	Category	Frequency	Percentage
Age at first pregnancy	≤18 years	368	92.5
	>18 years	30	7.5

Gravidity	Primigravida	235	59.0
	Multigravida	163	41.00

Parity	Primipara	276	69.3
	Multipara	122	30.7

Neonatal death	Yes	1	0.3
	No	397	99.7

Stillbirth	Yes	3	0.8
	No	395	99.2

History of abortion	Yes	18	4.5
	No	380	95.5

Labouring time	≤6 hours	259	65.1
	>6 hours	139	34.9

Travelling time to reach health facility	≤30 minutes	235	59.0
	>30 minutes	163	41.0

Time to be seen by the physician	≤20 minutes	294	73.9
	>20 minutes	140	26.1

Waiting time after delivery	≤24 hours	382	96.0
	>24 hours	16	4.0

ANC follow up	Yes	375	94.2
	No	23	5.8

Frequency of ANC follow up (n = 375)	<4 visits	230	61.3
	≥4 visits	145	38.7

Reason for institutional delivery	Recommended by others	160	40.2
	Satisfied with a previous birth	159	39.9
	Referred from other HFs	79	19.8

Status of pregnancy	Wanted	381	95.7
	Unwanted	17	4.3

Mode of delivery	SVD	365	91.7
	Instrumental delivery	16	4.0
	Caesarean section	17	4.3

Mode of transport	Walking	3	0.8
	Public transport	204	51.2
	Ambulance	191	48.0

Getting adequate meal	Yes	358	89.9
	No	40	10.1

**Table 3:** Factors associated with maternal satisfaction with intrapartum care among mothers who gave birth at a public hospital in North Wollo Zone, 2019 (*n* = 398).

Variable	Category	Maternal satisfaction		COR (95% CI)	AOR (95% CI)
		Satisfied No (%)	Dissatisfied No (%)		
Age	≤24	104 (65.0)	56 (35.0)	2.78 (1.17-6.61)	1.52 (0.38-6.17)
	25-34	89 (41.8)	124 (58.2)	1.07 (0.46-2.51)	0.49 (0.0.12-1.91)
	>34	10 (40.0)	15 (60.0)	1	1

Educational level	No formal education	154 (48.0)	167 (52.0)	0.39 (0.19-0.77)	0.99 (0.40-2.46)
	Primary education	18 (54.5)	15 (45.5)	0.50 (0.19-1.29)	1.15 (0.37-3.53)
	Secondary education and above	31 (70.5)	13 (29.5)	1	1

Pregnancy status	Wanted	197 (51.7)	184 (48.3)	1.96 (0.71-5.41)	1.63 (0.50-5.28)
	Unwanted	6 (35.3)	11 (64.7)	1	1

Parity	Primipara	189 (53.5)	164 (46.5)	1	1
	Multipara	14 (31.1)	31 (68.9)	0.39 (0.20-0.76)	0.54 (0.24-1.22)

Residence	Rural	68 (68.0)	32 (32.0)	2.57 (1.59-4.14)	**2.03 (1.05-3.93)** ^∗^
	Urban	135 (45.3)	163 (54.7)	1	1

Gravidity	Primigravida	163 (54.5)	136 (45.5)	1	1
	Multigravida	40 (40.4)	59 (59.6)	0.57 (0.36-0.89)	1.36 (0.70-2.64)

Mode of delivery	SVD	196 (53.7)	169 (46.3)	1	1
	Operative delivery	7 (21.2)	26 (78.8)	0.23 (0.09-0.54)	0.74 (0.22-2.37)

Time to be seen by HCP	≤20 minutes	168 (57.1)	126 (42.9)	2.63 (1.65-4.20)	**2.82 (1.46-5.46)** ^∗∗^
	>20 minutes	35 (33.7)	69 (66.3)	1	1

Getting adequate meal	Yes	194 (54.2)	164 (45.8)	4.07 (1.89-8.80)	**3.96 (1.13-13.83)** ^∗∗∗^
	No	9 (22.5)	31 (77.5)	1	1

ANC follow-up	Yes	183 (48.8)	192 (51.2)	1	1
	No	18 (78.3)	5 (21.7)	3.78 (1.65-14.77)	**3.73 (1.12-12.57)** ^∗∗∗∗^

Waiting after delivery	≤24 hrs.	173 (55.6)	138 (44.4)	2.38 (1.45-3.91)	1.44 (0.80-2.58)
	>24 hrs.	30 (34.5)	57 (65.5)	1	1

Significant at ^∗^*P* = 0.035, ^∗∗^*P* = 0.002, ^∗∗∗^*P* = 0.031, and ^∗∗∗∗^*P* = 0.034.

## Data Availability

All related data has been presented within the manuscript. The dataset supporting the conclusions of this article is available from the authors on request.

## Abbreviations

AOR: Adjusted odds ratio
ANC: Antenatal care
COR: Crude odds ratio
EDHS: Ethiopia demographic health survey
MMR: Maternal mortality ratio
PHCU: Primary health care units
SPSS: Statistical package for social science
SVD: Spontaneous vaginal delivery
WDU: Woldia University
WHO: World Health Organization.
